# Differences between homicide and filicide offenders; results of a nationwide register-based case-control study

**DOI:** 10.1186/1471-244X-9-27

**Published:** 2009-05-29

**Authors:** Hanna Putkonen, Ghitta Weizmann-Henelius, Nina Lindberg, Markku Eronen, Helinä Häkkänen

**Affiliations:** 1 Vanha Vaasa hospital, PO Box 13, 65381 Vaasa, Finland; 2 Helsinki University Central Hospital, Department of Adolescent Psychiatry, PO Box 590, 00029 HUCH, Hesinki, Finland; 3 Department of Psychology, PO Box 9, 00014 University of Helsinki, Helsinki, Finland; 4 National Bureau of Investigation, Forensic Laboratory, PO Box 285, 01301, Vantaa, Finland

## Abstract

**Background:**

Filicide, the killing of one's child, is an extraordinary form of homicide. It has commonly been associated with suicide and parental psychiatric illness. In the research on filicide, nationwide studies with comparison groups, specific perpetrator subgroups, and assessment of possible risk factors have been called for. The purpose of the current study was to provide all that.

**Methods:**

In this nationwide register-based case-control study all filicide offenders who were in a forensic psychiatric examination in Finland 1995–2004 were examined and compared with an age- and gender matched control group of homicide offenders. The assessed variables were psychosocial history, index offence, and psychiatric variables as well as psychopathy using the PCL-R.

**Results:**

Filicide offenders were not significantly more often diagnosed with psychotic disorders than the controls but they had attempted suicide at the crime scene significantly more often. Filicide offenders had alcohol abuse/dependence and antisocial personality less often than the controls. Filicide offenders scored significantly lower on psychopathy than the controls. Within the group of filicide offenders, the psychopathy items with relatively higher scores were lack of remorse or guilt, shallow affect, callous/lack of empathy, poor behavioral controls, and failure to accept responsibility.

**Conclusion:**

Since filicide offenders did not seem significantly more mentally disordered than the other homicide offenders, psychiatry alone cannot be held responsible for the prevention of filicide. Extensive international studies are needed to replicate our findings and provide more specific knowledge in order to enhance prevention.

## Background

Filicide is defined as the act of a parent killing her/his child. The killing of a child younger than one year is commonly called infanticide; when committed within the first 24 hours of life it is neonaticide. Over the years, filicide has been studied at length under a number of different classifications. One of the classic systems is that by Resnick [1] proposing a classification of filicide as 1) altruistic, 2) acutely psychotic, 3) unwanted child, 4) accidental, and 5) spousal revenge. Several reviews on the matter have recently been published [2-6].

Fortunately, filicide is not a very frequent crime, rates vary from 0.6 per 100,000 children under 15 to 2.5 per 100,000 children under 18 [7,8]. Yet, as is devastatingly clear, each case is highly disturbing. Moreover, rates of child murder are considered underestimates, due to inaccurate coroner rulings and some bodies never being discovered [9,10]. Filicide is an extraordinary form of homicide. In fact, it has been noted that rates of infanticide parallel suicide rates more closely rather than murder rates [11]. Indeed, suicide is commonly associated with filicide, both attempted and fulfilled suicide [2,5,8]. Contrasting findings disagree as to who commits filicide more often, mothers or fathers [1,8,12,13]. Furthermore, numerous studies indicate an association between filicide and parental psychiatric illness, namely major depression with psychotic features [1,2,5,14]. In addition, personality disorders, particularly borderline personality, have been found frequent in both female and male filicide offenders [2,15].

Psychopathy is an important construct in explaining criminal behavior and has especially been linked to violent criminality [16]. Characteristics of psychopathy form a particular pattern of interpersonal, affective, and behavioral symptoms. Egocentricity and impulsivity, lack of empathy and remorse, as well as shallow and labile affects are typical personality traits in psychopathy together with a violation of social norms [16]. The most widely used operational definition of psychopathy has been the Hare Psychopathy Checklist – Revised [16]. Although research on psychopathy is quite extensive, to our knowledge, no previous studies on psychopathy of filicide offenders exist. Yet, some of the psychopathic traits, e.g. egocentricity and lack of empathy might underlie the act of killing one's own children.

In spite of active research on filicide, nationwide studies are scarce. Moreover, since we have studied Finnish homicide offenders quite extensively [17-19], we have come to the conclusion that even though in Finland most homicide offenders, regardless of gender, are substance abusing and personality disordered, there might be subgroups of homicide offenders with a different history and psychiatric morbidity. We suspected that filicide offenders form such a subgroup. The aim of the present nationwide study was to compare the psychosocial history, index offence, and psychiatric morbidity of filicide offenders with those of other homicide offenders. Furthermore, we wanted to compare the prevalence of psychopathy and the discriminating value of the individual items of psychopathy between these groups.

## Methods

The National Authority for Medicolegal Affairs (NAMA) and the Ministry of the Interior approved the study procedures.

### Subjects

The material of the present study was register-based, comprehensive, and nationwide. It consisted of all the forensic psychiatric examination reports in Finland for offenders accused of homicide between 1995 and 2004 (N = 749). These 749 offenders were prosecuted for 700 homicidal events with a total of 757 victims. For the present study, cases of filicide (n = 25, 3.5% of homicides) were identified and gathered for data analyses. There were 25 child victims and 20 offenders. Eleven offenders were prosecuted for murder and nine for manslaughter. There were 14 cases with one victim, five with two victims (two with the partner and the child) and one with four victims (3 children and the partner). Sixteen of the victims were girls, nine were boys. The mean age of victims was four years (SD 4, range 0–13). Seven (28%) children were less than a year old. There were 13 female offenders and seven male. Two men were stepfathers but in an actual parental relationship with the child, all the rest were biological parents. There was a single offender in all cases. The mean age of all filicide offenders was 36 (SD 9, range 20–52). The female offenders' mean age was 25 (SD 8, range 20–45), while the male mean was 40 (SD 10, range 29–52). Of the 20 filicide offenders, 12 had lost custody of a child at some point in their lives.

Of the remaining offenders in the national data, a random sample of 20 age- and gender-matched offenders formed the comparison group. We allowed a case for comparison if there was a single perpetrator and the crime itself was with no extreme, exceptional features. In the comparison group, there were 5 murder and 15 manslaughter cases. Of the victims, 15 were acquaintances, 18 were male, two female. The mean age of the victims was 47 years (SD 14, range 24–75). Of the comparison offenders, 12 (60%) were parents of at least one child.

### Data sources

Among Western European nations, Finland ranks high in rates of homicide; in 2005, the total rate per 100,000 inhabitants of homicidal crimes reported to the police was 2.5 [20]. In the US, the homicide figure for 2005 was 5.6 per 100,000 [21]. The rate of children less than one year killed in homicide has decreased in Finland since the latter half of the 1990's. Between 2000–2005 it was 0.8 per 100,000 while within the age group 1–4 years, the figure was 0.9 per 100,000 [22]. Also in the US, homicides of children younger than one has declined since 1990 [21].

In Finland, the mean clearance rate for homicide was 92% between 1995–2004 [23]. Very few victims of homicide remain unknown to the police [24]. Furthermore, it is estimated that 85% of homicide offenders go through a forensic psychiatric examination as part of the trial procedure [25]. Because of the nature of the crime, it can be assumed that an even larger proportion of filicide offenders are examined. According to Finnish law, the courts decide if a forensic psychiatric examination is needed. After deciding on the examination, the court asks the NAMA to arrange the procedures. Forensic psychiatric examinations are inpatient evaluations lasting six weeks on average, and include data gathered from various sources (relatives as well as medical, criminal, school and military records), psychiatric evaluation, standardized psychological tests, interviews by a multi-professional team, physical evaluation, and continuous observation by hospital staff. The final forensic psychiatric report includes an evaluation of the level of criminal responsibility, a possible psychiatric diagnosis, and an assessment as to whether or not the offender fulfils the criteria for involuntary psychiatric care. All of the above are made by the forensic psychiatrist. Diagnoses made during the examinations were based on DSM-III-R criteria until 1996, when ICD-10 became the official classification. In addition, DSM-IV has been widely used. Previous studies have successfully reported across this margin of classifications [18,26]. The NAMA instructs and controls the standards of the examinations, and overall quality and reliability of Finnish forensic psychiatric examinations are considered high by both courts and scientists [27]. All reports for the current study were carefully analyzed for variables related to psychosocial and mental health issues: offenders' parents' alcohol/mental health problems or criminal history, and their socio-economic status, offenders' adulthood socio-economic variables (marital status, criminality, employment status), use of mental health services, suicidal behavior, and the results of the forensic psychiatric examination, including the psychiatric diagnoses. Interrater reliability of the crime scene behavior and offender background variables has been assessed in our previous studies, with partly the same data and data collection procedure [18,26]. Thus, a random sample of 18 cases were picked and coded by two researchers and subjected to Cohen's kappa analysis. With continuous variables, Pearson's correlation coefficient was used. When the amount of agreement was of statistical significance (p < .05), the variable was included in further statistical analysis [28]. The reports were gathered from the NAMA's archives.

*The Hare Psychopathy Checklist-Revised *[16] is a 20-item rating scale based on a semi-structured interview and a review of collateral information. The items are rated on a three-point scale according to the degree to which the personality and behavior matches the item description. The total score amounts to 40. In addition to the diagnostic cut-off score of 30, recommended by Hare [16], a second cut-off score of 25 is often used in European studies [29,30]. In this European study, we reported both. The revised scale has a two-factor structure; the interpersonal/affective and social deviance and four facets: interpersonal, affective, lifestyle and antisocial [16]. Cooke and Michie [31] have proposed a three-factor hierarchical model, which measures the superordinate factor of psychopathy, which is underpinned by three factors: the arrogant and deceitful interpersonal style, deficient affective experience, and impulsive and irresponsible behavioral style. In the current study, forensic evaluation reports of the offenders were reviewed and the PCL-R was retrospectively rated by trained raters. Studies have shown that file-only PCL-R ratings can be used for research purposes with solid reliability. The findings by Grann [30] support the use of file-based ratings for research purposes calculating ICC(2,1) = .88 for the total scores, ICC(2,1) = .69 for factor 1 and ICC(2,1) = .89 for factor 2. Also Wong [32] has calculated interrater reliability: Pearson r = .74. Both groups suggested that ratings on the PCL-R should only be performed without an interview if comprehensive file information is available. Our ratings were based on reviewing a very comprehensive set of objective institutional files consisting of reports from many different disciplines.

To evaluate inter-rater agreement of the PCL-R further, 20 reports were randomly chosen from the total national data and rated by all raters after preparation in workshop attendance and several training sessions. The inter-rater agreement was assessed using Intraclass correlation ICC_(2,1). _The ICC was .898 for the PCL-R total score; .735 for factor 1 and .920 for factor 2 scores. All correlations were significant (*p *< .001). The internal consistency, as measured by Cronbach's alpha, was .89 for all items, .86 for factor 1 and .79 for factor 2, .84 for facet 1/factor 1 in the three factor model, .83 for facet 2/factor 2 in the three factor model, .87 for facet 3/factor 3 in the three factor model, and .64 for facet 4.

### Statistical analysis

Data analyses were made with SPSS 16.0 statistical software package. Chi-square analysis and Fisher's Exact Test were used to compare differences in frequencies between the groups. Differences in mean PCL-R scores were assessed by Mann-Whitney U-Test.

## Results

Five (25%) filicide offenders were diagnosed with a psychotic disorder whereas two (10%) of the other homicide offenders were. The difference was not statistically significant. In both groups there were two offenders with schizophrenia but there were three filicide offenders with psychotic depression. Ten filicide offenders and 12 of the other homicide offenders had previously received psychiatric outpatient treatment. Other differences are shown in Table 1.

**Table 1:** Case-control comparison of filicide offenders 1995–2004

	**Filicide** **(N = 20)**		**Comparison** **(N = 20)**		p*
			
	N	%	N	%	
**Index offence**					
Stabbing	4	20	16	80	0.000
Intoxicated with alcohol	8	40	16	80	0.017
Attempted suicide immediately after	10	50	0	0	0.000
Motive – quarrel	2	10	10	50	0.014
**Psychosocial History**					
Previous offending	4	20	13	65	0.010
Violent offending	3	15	9	47	0.041
Property offending	4	20	12	60	0.022
Previous psychiatric hospitalization	6	30	13	65	0.056
Previous documented suicidal behavior	8	40	9	45	ns
Employed at the time of offence	10	53	3	15	0.019
Biological parent of any child	19	95	12	60	0.020
**Forensic Psychiatric examination**					
Personality disorder	11	55	15	75	ns
Borderline personality	2	10	4	20	ns
Antisocial personality	1	5	7	35	0.044
Alcohol abuse/dependence (a/d)	4	21	14	70	0.004
Personality disorder and Alcohol a/d	2	10	12	60	0.002
					
Full criminal responsibility	5	25	14	70	0.010
Diminished criminal responsibility	10	50	4	20	0.096
No criminal responsibility	5	25	2	10	ns

* Fisher's Exact test, two- tailed, ns = not significant

The mean PCL-R score, prorated for missing items for filicide offenders was 10.2 (SD 7.5, range 0–28.4) and for the comparison group 21.9 (SD 8.6, range 3.2–35.8). The difference was significant, U_(1) _= 66.5, p < 0.001. None of the filicide offenders received a score of 30 or over, but three (15%) of the comparison group did. Of the filicide offenders two (10%) received a PCL-R score of 25 or more while seven (35%) of the comparison group did.

The mean for factor 1 score in the filicide offender group was 5.3 (SD 3.5, range 0–16) and in the comparison group 10.0 (SD 3.6, range 3–16). The mean for factor 2 score in the filicide offenders was 4.1 (SD 4.6, range 0–16) and in the comparison group 10.1 (SD 5.6, range 0–18). The differences were significant (factor 1 score U_(1) _= 67, p < 0.001 and factor 2 score U_(1) _= 80, p < 0.002). There was also a significant difference between the filicide and comparison groups in the mean PCL-R scores of the three-factor model. Items indicating antisocial behavior are excluded from this model. The mean scores were 7.6 (SD 5.4) in the filicide group and 16.7 (SD 5.8) in the comparison group (U_(1) _= 55, *p *< 0.001).

There were significant differences between the filicide and the comparison groups in 10 of the 20 individual PCL-R items as shown in Table 2.

**Table 2:** PCL-R scores item-by-item – Filicide offenders and comparison group (N = 20 in both groups)

	**Filicide-** **group**	**Comparison-** **group**	p *
			
**Item**	**%**	**%**	
**1. Glibness/superficial charm**			
score 0	80.0	65.0	ns
scores 1 and 2	20.0	35.0	
**2. Grandiose sense of self worth**			
score 0	70.0	35.0	ns
scores 1 and 2	30.0	65.0	
**3. Need for stimulation**			
score 0	80.0	20.0	.001
scores 1 and 2	20.0	80.0	
**4. Pathological lying**			
score 0	95.0	55.6	.007
scores 1 and 2	5.0	44.4	
**5. Conning/manipulative**			
score 0	75.0	31.6	.010
scores 1 and 2	25.0	68.4	
**6. Lack of remorse or guilt**			
score 0	35.0	10.0	ns
scores 1 and 2	65.0	90.0	
**7. Shallow affect**			
score 0	25.0	0.0	.047
scores 1 and 2	75.0	100.0	
**8. Callous/lack of empathy**			
score 0	45.0	5.0	.008
scores 1 and 2	55.0	95.0	
**9. Parasitic lifestyle**			
score 0	65.0	40.0	ns
scores 1 and 2	35.0	60.0	
**10. Poor behavioral controls**			
score 0	45.0	20.0	ns
scores 1 and 2	55.0	80.0	
**11. Promiscuous sexual behavior**			
score 0	66.7	66.7	ns
scores 1 and 2	33.0	33.3	
**12. Early behavior problems**			
score 0	80.0	68.4	ns
scores 1 and 2	20.0	31.6	
**13. Lack of realistic goals**			
score 0	75.0	10.5	.001
scores 1 and 2	25.0	89.5	
**14. Impulsivity**			
score 0	60.0	15.0	.008
scores 1 and 2	40.0	85.0	
**15. Irresponsibility**			
score 0	55.0	10.0	.006
scores 1 and 2	45.0	90.0	
**16. Failure to accept responsibility**			
score 0	26.3	0.0	.020
scores 1 and 2	73.7	100.0	
**17. Many short-term marital relationships**			
score 0	73.7	61.1	ns
scores 1 and 2	26.3	38.9	
**18. Juvenile delinquency**			
score 0	95.0	77.8	ns
scores 1 and 2	5.0	22.2	
**19. Revocation of conditional release**			
score 0	88.9	61.5	ns
scores 1 and 2	11.1	38.5	
**20. Criminal versatility**			
score 0	89.5	55.0	.031
scores 1 and 2	10.5	45.0	

* Chi-square test, df = 2, two tailed, ns = not significant

## Discussion

The novel results of this nationwide register-based case-control study reinforced the general finding that filicide offenders are a distinct group of homicide offenders. However, they did not emerge as mentally disordered as previously supposed. We found that filicide offenders score significantly lower on the PCL-R in comparison with other homicide offenders.

Filicide offenders were not as often intoxicated with alcohol during the crime and they had significantly less previous criminal offending than the homicide controls. They were, furthermore, more often employed than the matched comparison group. This clearly contrasts with the largest group of Finnish homicide offenders, the substance-abusing (mostly alcohol), impulsive, marginalized, and antisocial men [22,33]. The filicide offenders seemed more socially and societally conformed than the other offenders, a finding which had been previously noted [12,34].

There were not many differences between the groups regarding psychiatric diagnoses. Contrary to what might have been expected based on previous research, the prevalence of psychotic disorders did not statistically significantly differ between the groups. However, there were three filicide offenders with psychotic depression. This finding might have clinical significance, since it is the diagnosis previously found prevalent in filicide offenders [35] and filicide-suicide offenders [4]. Half of our filicide offenders but none of the controls attempted suicide on the crime scene. The association between filicide and suicide has been shown before [2,5]. It seems filicide as a phenomenon is closely associated with suicide; perhaps at times it is even more about the suicide than the homicide. Moreover, alcohol abuse/dependence and antisocial personality disorder as well as psychopathy were more frequent among the other homicide offenders. In spite of all the above, criminal responsibility was assessed lower in the filicide group. Perhaps there are issues related to filicide not illustrated in diagnostics but displayed in the responsibility assessment. It might be emotionally challenging to consider someone to have killed a child in a completely responsible state of mind.

The filicide offenders were not psychopaths. There were no previous studies on psychopathy within filicide populations so this result must await further confirmation. The low prevalence of psychopathy among these female and male filicide offenders was in line with the results by Warren et al. [36], which would indicate that filicide is mostly a homicide similar to murder. Warren et al. [36] found lower prevalence of psychopathy among women guilty of murder than of other violent crimes. Both Warren's murderers and our filicide offenders had previous offending less often than the control groups. In the present study, none of the filicide offenders scored 30 or more on the PCL-R, and only two (10%) 25 or more. The prevalence of psychopathy using the cut-off score of 30 or more usually falls between 9 and 23% in female and 15 and 30% in male offender samples [37].

The filicide offenders scored significantly lower than the comparison group on ten PCL-R items. It is important to note, however, that the significant differences were not on the "antisocial" items, with the exception of criminal versatility. Nevertheless, it is noteworthy that more than half of the filicide offenders scored 1 or 2 on the items lack of remorse or guilt, shallow affect, callous/lack of empathy, poor behavioral controls, and failure to accept responsibility. All of these except poor behavioral control compose the Cooke's factor deficient affective experience and characterized the filicide offender as a person with emotional dysfunction [38]. Hence, the majority of filicide offenders may represent the same constellation of personality traits found in domestic batterers [39]. Emotional deficiency may predispose to violent behavior when the person cannot emotionally consider the harmful consequences of her/his actions [40]. A further interesting finding was the lack of significant difference in the prevalence of borderline personality disorder. This might show that the PCL-R items measure a different type of emotional dysfunction – more incapability than volatility of emotions.

### Strengths and limitations

Filicide has been a challenging topic for research because of small data. Hatters Friedman et al. [3] noted that the nature of filicide varies within the study populations and suggested that further studies include, e.g., comparison groups, specific perpetrator subpopulations, and national populations. In this study we achieved just that.

This was a unique nationwide, comprehensive study which is a definite strength. We studied all the forensic psychiatric examinations for homicide in Finland between 1995–2004. The Finnish clearance rate for homicide and the tradition of thorough forensic psychiatric examinations and statistics form a solid basis for a register-based study; one can even suppose that the studied offenders were fairly representative of Finnish filicide offenders. Most Finnish homicide offenders are examined thoroughly and it can be presumed, given the graveness of the crime, that even a larger percentage of filicide offenders are examined. The diagnoses of the Finnish forensic psychiatric examinations are always based on exhaustive examination and specific criteria. Therefore they are considered reliable for study purposes [17,19]. However, the fact that the present study was retrospective and register-based does present some obvious limitations, though the same limitations apply both the filicide and the comparison group. Clearly, the small number of cases limits analyses but given the rarity of filicide large numbers are unattainable even in national samples.

Since filicide is a crime which raises many emotions, it has to be considered whether or not scorers' attitudes might have affected scoring. We did not find this a considerable problem since the scorers only received the information in the forensic psychiatric examination reports, i.e. objective statements for court procedures, and, furthermore, the control group also comprised serious offenders. What's more, bias issues were attended to in training. Grann et al. [30] has stated that given how little we know about gender differences and gender bias, it is essential that these issues be specifically addressed during PCL-R training. This must be true for any biased assumptions.

## Conclusion

Contrary to previous conclusions, we did not find that the filicide offenders had significantly more mental illness and more serious psychopathology than the other homicide offenders. Psychopathy was certainly not a risk factor for filicide. However, the filicide offender did exhibit emotional problems which should be noted as risk factors and suicidal behavior at the crime scene might signal distress unlike that of the common homicide offender. The filicide offenders might be incapable of handling even everyday difficulties. We therefore conclude that prevention of filicide cannot remain the task of psychiatry alone, but health care and society at large must work to forestall the danger of filicide. Parents who are severely fatigued or otherwise not able to cope should receive adequate support. However, mental health services cannot relax, and our results should be replicated. In order to find in-depth information on filicide and associated social, emotional, and psychopathological issues and, therefore, to enhance prevention, we urgently need international cooperation to study filicide in a large scale database.

## Competing interests

Dr. Lindberg has received travel funds from Novartis and Bristol-Myers Squibb during 2008. Dr. Eronen has received speakers honoraria from Bristol-Myers Squibb, Astra Zeneca, Orion and Novartis, and Dr Häkkänen from Bristol-Myers Squibb. These are single occasions with minor economic significance. All other authors report no competing interests.

## Authors' contributions

HP contributed to conception, design, and acquisition of data, analyzed and interpreted data, and served as first author. GW-H contributed to conception, design, and acquisition of data, analyzed and interpreted data, and participated in the writing process. NL contributed to conception, design, and acquisition of data, and participated in the writing process. ME contributed to conception, design, and participated in the writing process.HH provided the material, contributed to conception, design, and acquisition of data, and participated in the writing process.

## Pre-publication history

The pre-publication history for this paper can be accessed here:


